# Supplementary material for: Branched Chain Amino Acid Supplementation to a Hypocaloric Diet Does Not Affect Resting Metabolic Rate but Increases Postprandial Fat Oxidation Response in Overweight and Obese Adults after Weight Loss Intervention
Source: Nutrients. 2021 Nov 26;13(12):4245. doi: 10.3390/nu13124245 (PMC8708242; doi:10.3390/nu13124245)
Supplement: Supplementary file 1 [file nutrients-13-04245-s001.zip › nutrients-1416691-supplementary.pdf]

Table S1. Baseline characteristics of study participants in the three diet groups

Parameters	CT (n=37)	BCAA (n=35)	HP (n=39)	*p*
Age (years)	34.7±7.77	32.0±6.79	31.1±6.53	0.072
Sex (Male/Female)	18/19	17/18	20/19	0.965
Weight (kg)	80.6±10.9	83.93±12.71	82.2±13.08	0.507
BMI (kg/m^2)	29.4±2.31	29.8±2.69	29.7±3.14	0.860
Waist circumference (cm)	94.1±7.58	96.0±6.82	96.4±8.42	0.391
Body fat percentage (%)	39.2±5.47	39.2±5.78	38.9±5.57	0.967
Total body fat mass (kg)	30.9±5.20	32.1±5.60	31.1±5.16	0.601
Total body lean mass (kg)	45.9±8.59	47.9±9.82	47.2±10.1	0.656
Systolic blood pressure (mmHg)	119±10	116±10	117±9	0.288
Diastolic blood pressure (mmHg)	77±10	75±6	77±7	0.519
RMR (kcal/day)	1570±238	1610±273	1600±300	0.753
Fat oxidation (%)	54.5±22.1	58.4±17.0	53.7±22.9	0.588
Carbohydrate oxidation (%)	46.0±22.1	42.1±17.0	46.8±22.9	0.593
RQ	0.84±0.08	0.83±0.05	0.84±0.07	0.608
Total cholesterol (mmol/L)	4.83±0.66	4.63±0.61	4.77±0.77	0.442
Triglycerides (mmol/L)	1.06±0.39	1.18±0.55	1.16±0.48	0.537
HDL cholesterol (mmol/L)	1.24±0.18	1.25±0.32	1.20±0.25	0.625
LDL (mmol/L)	3.10±0.56	2.84±0.55	3.05±0.61	0.139
Fasting glucose (mmol/L)	4.90±0.38	4.78±0.32	4.85±0.42	0.383
Fasting insulin (mU/L)	8.44±4.31	8.58±5.80	7.93±4.29	0.829
HOMA-IR	1.88±1.09	1.82±1.19	1.73±0.99	0.820

Data presented as mean ± SD or N. One-way ANOVA with Bonferroni correction and Chi-square test were used to compare differences in continuous and categorical parameters respectively, among the 3 diet groups.

Abbreviations: Body mass index, BMI; Resting metabolic rate, RMR; Respiratory quotient, RQ; High-density lipoprotein, HDL; Low-density lipoprotein, LDL; Homeostatic model assessment of insulin resistance, HOMA-IR.

Table S2. Clinical characteristics of study participants before (week 0) and after (week 16) dietary weight loss intervention

Parameters	Week 0 (N=111)	Week 16 (N=111)	*p*
Age (years)	32.6±7.15	32.6±7.15	-
Sex (male/female)	55/56	55/56	-
Weight (kg)	82.2±12.2	75.7±12.4	<0.0005
BMI (kg/m^2)	29.6±2.72	27.3±2.86	<0.0005
Waist circumference (cm)	95.5±7.66	88.6±7.72	<0.0005
Body fat percentage (%)	39.1±5.55	36.6±5.95	<0.0005
Total body fat mass (kg)	31.4±5.29	27.1±5.79	<0.0005
Total body lean mass (kg)	47.0±9.49	44.9±9.19	<0.0005
Systolic blood pressure (mmHg)	117±10	114±10	<0.0005
Diastolic blood pressure (mmHg)	77±8	73±10	<0.0005
RMR (kcal/day)	1600±270	1500±264	<0.0005
Fat oxidation (%)	55.4±20.8	55.6±20.1	0.950
Carbohydrate oxidation (%)	45.1±20.8	44.9±20.1	0.950
RQ	0.84±0.07	0.84±0.07	0.930
Total cholesterol (mmol/L)	4.74±0.68	4.43±0.73	<0.0005
Triglycerides (mmol/l)	1.13±0.47	0.96±0.38	<0.0005
HDL cholesterol (mmol/L)	1.23±0.5	1.16±0.24	<0.0005
LDL cholesterol (mmol/L)	3.00±0.58	2.84±0.61	<0.0005
Fasting glucose (mmol/L)	4.85±0.38	4.68±0.36	<0.0005
Fasting insulin (mU/L)	8.30±4.79	4.95±2.67	<0.0005
HOMA-IR	1.81±1.08	1.04±0.58	<0.0005

Data presented as mean ± SD or N. Paired t-test and Chi-square test were used to compare differences in continuous and categorical parameters respectively, between week 0 and week 16.

Abbreviations: Body mass index, BMI; Resting metabolic rate, RMR; Respiratory quotient, RQ; High-density lipoprotein, HDL; Low-density lipoprotein, LDL; Homeostatic model assessment of insulin resistance, HOMA-IR.

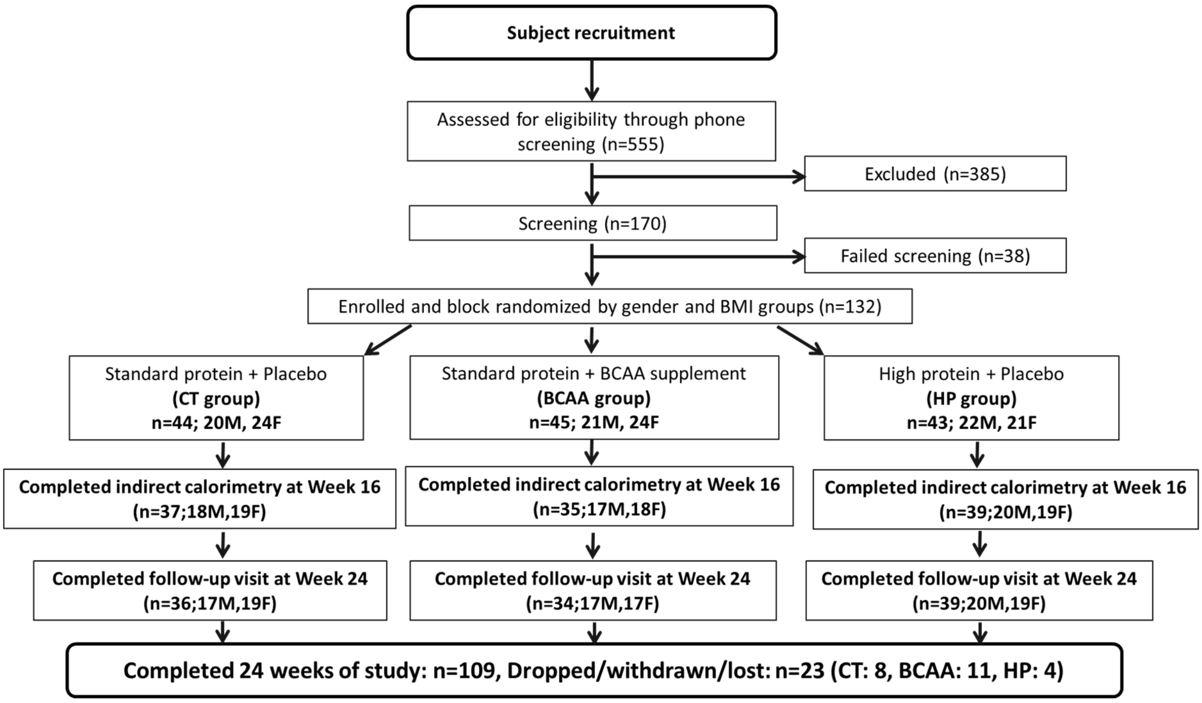

Figure S1. Consort diagram for recruitment, screening, enrolment and randomization of study subjects. One hundred and eleven subjects (55 men, 56 women) completed indirect calorimetry after 16 weeks of diet intervention. One hundred and nine subjects (54 men, 55 women) completed the 24-week intervention study. Twenty-three subjects did not complete the study: 1 subject was uncontactable for her last study visit at week 24; 8 subjects self-withdrew as they were unable to comply with our strict diet plan and 3 self-withdrew due to inability to commit to study visits (because of family, work or personal commitments); 4 subjects were dropped because they achieved <1.8kg weight loss at week 4 which is indicative of poor compliance, 6 were withdrawn by the principal investigator (PI) for health or medical reasons not directly related to this intervention and 1 was withdrawn by the PI for non-compliance with diet**.** M = male, F= female**.**

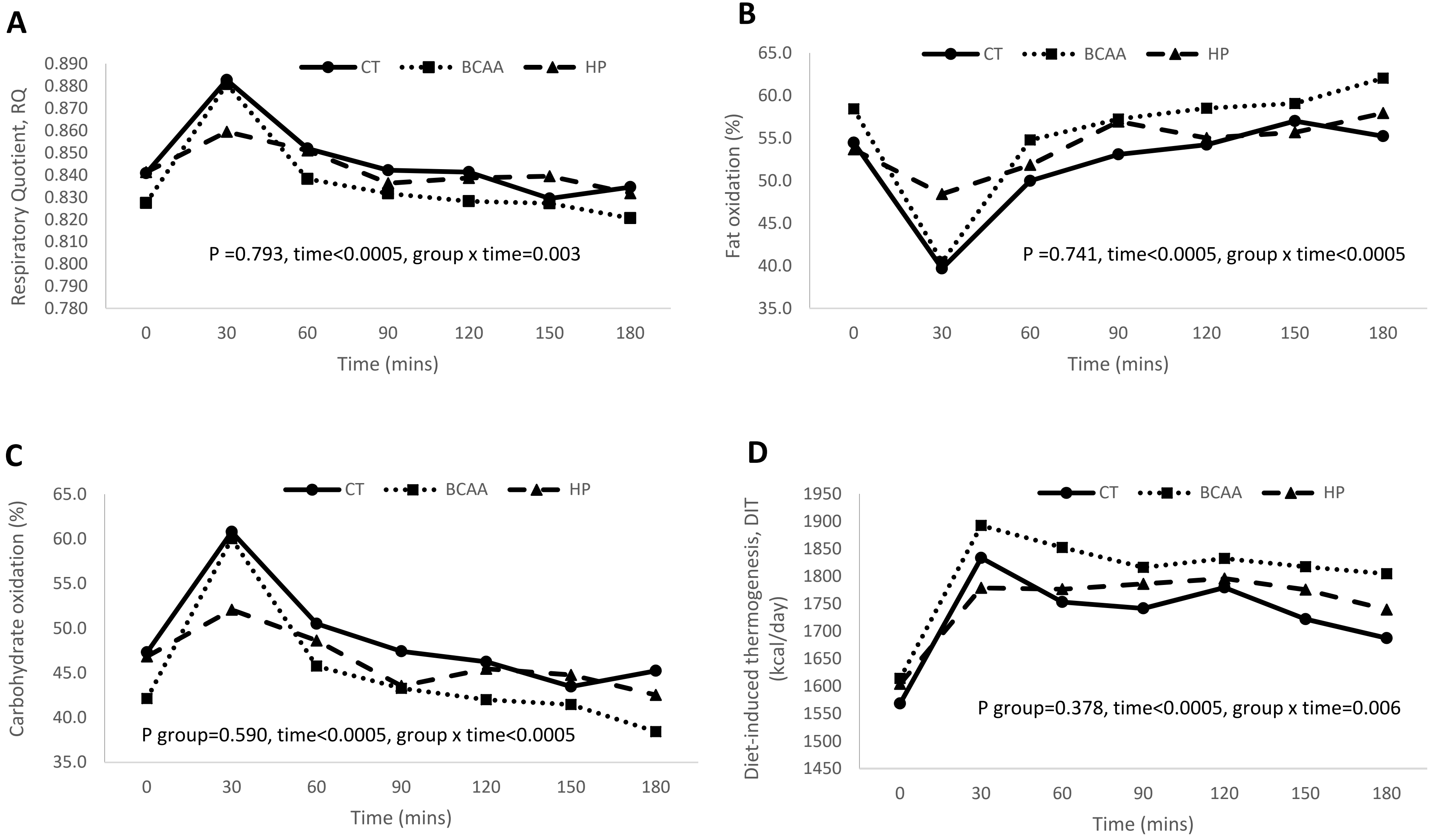
A
CT
BCAA
HP
Respiratory Quotient, RQ
0.890
0.880
0.870
0.860
0.850
0.840
0.830
0.820
0.810
0.800
0.790
0.780
P =0.793, time<0.0005, group x time=0.003
0
30
60
90
120
150
180
Time (mins)
B
CT
BCAA
HP
Fat oxidation (%)
65.0
60.0
55.0
50.0
45.0
40.0
35.0
P =0.741, time<0.0005, group x time<0.0005
0
30
60
90
120
150
180
Time (mins)
C
CT
BCAA
HP
Carbohydrate oxidation (%)
65.0
60.0
55.0
50.0
45.0
40.0
35.0
P group=0.590, time<0.0005, group x time<0.0005
0
30
60
90
120
150
180
Time (mins)
D
CT
BCAA
HP
Diet-induced thermogenesis, DIT (kcal/day)
1950
1900
1850
1800
1750
1700
1650
1600
1550
1500
1450
P group=0.378, time<0.0005, group x time=0.006
0
30
60
90
120
150
180
Time (mins)

Figure S2. Postprandial RQ, fat oxidation, carbohydrate oxidation and DIT responses among the three diet groups at baseline (Week 0).

A: Postprandial RQ responses among the 3 diet groups; B: Postprandial fat oxidation responses among the 3 diet groups; C: Postprandial carbohydrate oxidation responses among the 3 diet groups; D: Postprandial DIT responses among the 3 diet groups. Data was analysed by repeated measures ANOVA (between subject groups)

Abbreviations: Standard-protein with BCAA, BCAA; Standard-protein with placebo, CT; Diet-induced thermogenesis, DIT; High-protein with placebo, HP; Respiratory quotient, RQ
